# Breeding response of transcript profiling in developing seeds of *Brassica napus*

**DOI:** 10.1186/1471-2199-10-49

**Published:** 2009-05-24

**Authors:** Yaping Hu, Gang Wu, Yinglong Cao, Yuhua Wu, Ling Xiao, Xiaodan Li, Changming Lu

**Affiliations:** 1 Oil Crops Research Institute, Chinese Academy of Agricultural Sciences, No. 2 Xudong 2nd Road, Wuhan, 430062, PR China

## Abstract

**Background:**

The upgrading of rapeseed cultivars has resulted in a substantial improvement in yield and quality in China over the past 30 years. With the selective pressure against fatty acid composition and oil content, high erucic acid- and low oil-content cultivars have been replaced by low erucic acid- and high oil-content cultivars. The high erucic acid cultivar Zhongyou 821 and its descendent, low erucic acid cultivar Zhongshuang 9, are representatives of two generations of the most outstanding Chinese rapeseed cultivars (*B. napus*) developed the past 2 decades. This paper compares the transcriptional profiles of Zhongshuang 9 and Zhongyou 821 for 32 genes that are principally involved in lipid biosynthesis during seed development in order to elucidate how the transcriptional profiles of these genes responded to quality improvement over the past 20 years.

**Results:**

Comparison of the cultivar Zhongyou 821 with its descendent, Zhongshuang 9, shows that the transcriptional levels of seven of the 32 genes were upregulated by 30% to 109%, including *FAD3*, *ACCase, FAE1*, *GKTP*, *Caleosin*, *GAPDH*, and *PEPC*. Of the 32 genes, 10 (*KAS3, β-CT, BcRK6, P450, FatA, Oleosin, FAD6, FatB, α-CT *and *SUC1*) were downregulated by at least 20% and most by 50%. The *Napin *gene alone accounted for over 75% of total transcription from all 32 genes assessed in both cultivars. Most of the genes showed significant correlation with fatty acid accumulation, but the correlation in ZS9 was significantly different from that in ZY821. Higher *KCR2 *activity is associated with higher C16:0, C18:0, and C18:2 in both cultivars, lower C22:1 and total fatty acid content in ZY821, and lower 18:1 in ZS9.

**Conclusion:**

This paper illustrates the response of the transcription levels of 32 genes to breeding in developing rapeseed seeds. Both cultivars showed similar transcription profiles, with the *Napin *gene predominantly transcribed. Selective pressure for zero erucic acid, low glucosinolate, high oleic acid and high oil content, as well as high yield, resulted in higher *FAD3*, *ACCase, FAE1*, *GKTP*, *Caleosin*, *GAPDH*, and *PEPC *expression levels and lower *KAS3, β-CT, BcRK6, P450, FatA, Oleosin, FAD6, FatB, α-CT *and *SUC1 *expression levels. It also resulted in altered relationships between these genes during storage accumulation in seed development.

## Background

Rapeseed (*Brassica napus L.*) is one of the most important oil crops in the world. The annual planting acreage is about 8 MH(million hectare) in China, which accounts for a quarter of the world's total rapeseed production. Over the past 30 years, with the rape cultivar upgrades, China's rapeseed production has experienced tremendous advancement. The substantial improvement in yield and quality has greatly expanded the cultivation area and improved production efficiency in China.

Before 1990, almost all rapeseed cultivars in China were high in erucic acid and glucosinolate levels (double-high). Zhongyou 821 (abbrev. ZY821) dominated rapeseed planting along the middle and lower reaches of the Yangtze River at that time, with an annual planting area of around 1.8 MH in the late 1980s and early 1990s. With intense breeding toward high oil content and double low quality (low erucic acid and low-glucosinolate levels), Zhongshuang 9 (abbrev. ZS9), bred using ZY821 as one of the crossing parents, was released in 2002. Due to better comprehensive agronomic characteristics, ZS9 has rapidly replaced ZY821 and become one of the most popular double-low rapeseed cultivars in China. From 2003 to 2007, the total planting acreage of ZS9 was in excess of 0.47 MH. ZS9 is characterized by a 2.2% higher oil content, 51.1% higher oleic acid content, 43.4% lower erucic acid content and 8.6% lower eicosenoic acid content than ZY821 (Table 1). The double high cultivar ZY821 and the double low cultivar ZS9 have been two of the most popular rapeseed cultivars in China, representing two generations of Chinese rapeseed cultivars and reflecting the major achievements in rapeseed quality improvement over the past 30 years.

**Table 1:** Difference between ZY821 and ZS9.

	ZY821	ZS9	Difference
Yield (kg/ha)	9.57	11.03	
Protein content (%)	31.27%	32.83%	1.56% *
Oil content(%)	39.80%	42.00%	2.20% **
Glucosinolate content in meal(μm/g)	123.50	19.18	-104.32 **
Erucic acid (%)	42.00%	0.23%	-45.77% **
Eicosenoic acid (%)	9.75%	1.20%	-8.55% **
Oleic acid	16.90%	68.00%	51.10% **
Duration of dominating cultivation	1988–1995	2003-Now	
Average annual coverage	1.80 MH	0.47 MH	

*means different character, **means larger different character.

Significant improvements in oil quality must be accompanied by changes in gene activities involving fatty acid biosynthesis in developing seeds. The elucidation of gene transcription patterns associated with a specific stage of seed development is critical for understanding the molecular and biochemical events related to oilseed quality improvement. Characterization of these genes and their regulatory elements would provide not only new genetic information for understanding *B. napus *seed development, but also alternative seed promoters for controlling gene expression in developing seeds. Liu *et al*. analyzed the proteomic pattern and the gene transcripts of early germs with high-oil content and normal inbred lines in maize and found that three enzymes involved in lipid metabolism, namely, putative enoyl-ACP reductase (*ENR*), putative stearoyl-ACP desaturase (*SAD*) and putative Acetyl-CoA carboxylase (*ACCase*), had more abundant expression in high-oil lines than in the normal lines [1]. High expression of *SAD*, *ENR *and *ACCase *was associated with increasing oil concentration in high-oil maize. Dong *et al*. described the tissue-specific expression of 54 highly expressed mRNAs during early stages of seed development in *B. napus *and found that the majority of the seed-specific genes that are expressed at early stages of seed development encoded proteins with high similarity to hypothetical *Arabidopsis *proteins [2]. Genes in various metabolic and storage pathways exhibit distinct timing patterns, indicating that different sets of transcriptional factors turn on oil and protein pathway genes [3-5]. Knowledge of the expression of multiple genes and their regulation during oil biosynthesis is needed to further understand the regulatory mechanisms controlling oil metabolism [6]. This understanding will aid in the development of suitable tools for altering seed development and seed quality traits by molecular genetics.

Rapeseed seed quality in China has been drastically improved under selective pressure against fatty acid composition and oil content, but no reports have been made on the associated changes in related gene activities. In this study, we investigate how the transcriptional profiles of genes involved in fatty acid biosynthesis respond to the selective pressures against oil content and fatty acid composition and how the genes coordinate with each other. Our results provide a first glimpse of the gene expression and regulation breeding response to selective pressures in rapeseed, which is critical to metabolic engineering and the quality improvement of oilseeds.

## Results

### Seed Development and Fatty Acid Accumulation Patterns

During the first few weeks of seed development, the seed coat expanded until the seed was almost full size. At this stage, the seed was somewhat translucent and resembled a water-filled balloon. It was difficult to dissect ovules from the pod at 5 DAP(days after pollination) but it became possible after 10 DAP. The seed's embryo now began development and grew rapidly within the seed coat to fill the space previously occupied by fluid. The mature seed was harvested at 45 DAP.

**Figure 1:**
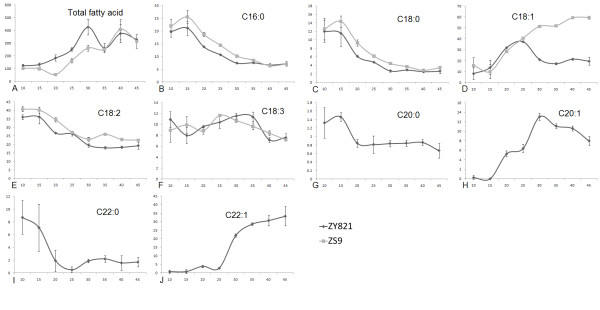
**Fatty acid accumulation patterns in seed development**. (A) The trend of total fatty acid accumulation in seed development. Fatty acids peaked at different times but the end accumulation was similar. (B-J) The accumulation pattern of fatty acids present in both cultivars. Most share similar accumulation patterns, but C18:1 was quite different between the two cultivars. Values in parentheses indicate the relative composition percentage and values in abscissa indicate the seed development stage. The black curves represent ZY821 and the gray curves represent ZS9. Each data point represents the mean ± SD of three replicates.

In the ZS9 cultivar, the total fatty acid content continued to increase and peaked at about 40 DAP, followed by a gentle drop with seed maturation (Fig. 1A). In contrast, ZY821 showed a similar pattern of accumulation of total fatty acids except that it had higher levels at 30 DAP (Fig. 1A). Nine fatty acids were found in ZY821 (Fig. 1B–J) but only five in ZS9 (Fig. 1B–F). Among the five common fatty acids, only oleic acid (C18:1) showed different accumulation patterns (Fig. 1D), whereas the other fatty acids accumulated in similar patterns in both cultivars (Fig. 1B–F). In ZS9, the oleic acid content increased continuously and became a major component at the end (Fig. 1D), whereas in ZY821, oleic acid ascended in the early stages (10–25 DAP) but decreased from 25 DAP to 35 DAP and then remained constant until the end (Fig. 1D). The initial content of all saturated fatty acids (C16:0, C18:0, C20:0 and C22:0) was relatively high but decreased from 15 DAP and reached a minimum at about 25–30 DAP, ultimately comprising a minor proportion of the fatty acids in mature seeds (Fig. 1B, C, G, I). The content of C18:2 was as high as 35–42% during the initial stages (10–15 DAP) and decreased continuously to a final content of about 20% (Fig. 1E). The C18:3 content fluctuated from 6% to 11.4% and was fixed at about 7% in mature seeds (Fig. 1F).

### Relative Transcript Levels for the 32 Genes

**Figure 2:**
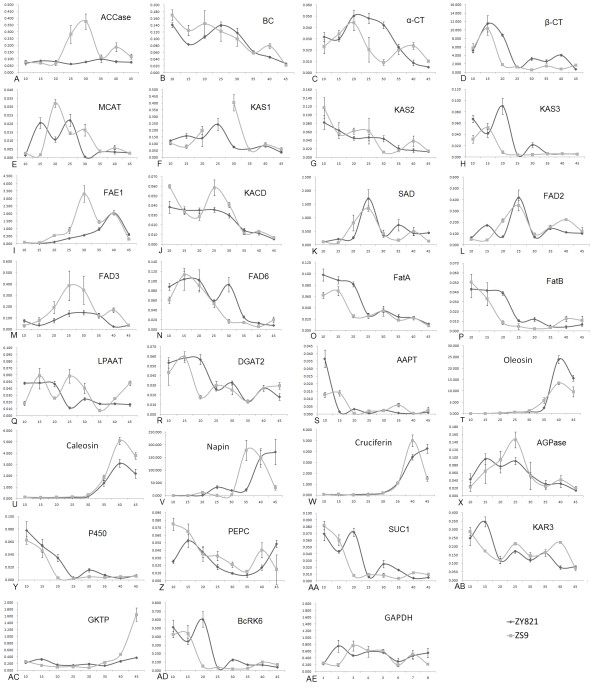
**The expression profile of fatty acid synthesis genes during seed development**. (A-D) ACCase gene family. (E-J) Genes involved in fatty acid elongation. (K-N) Desaturase. (O-P) Thioesterase. (Q-S) Genes involved in TAG synthesis. (T-W) Oil body proteins. (X-AE) Other basic metabolism genes. Abbreviated names for the genes are defined in Table 4. Values in parentheses indicate the relative copy number and Values in abscissa indicate the seed development stage. The black curves represent ZY821 and the gray curves represent ZS9. Each data point represents the mean ± SD of three replicates.

Different genes had different transcript levels but with very similar tendencies between the two cultivars in general (Table 2 and Fig. 2). In both cultivars, ZY821 and ZS9, the top 7 genes, including *Napin*, *Oleosin*, *β-CT*, *Caleosin*, *β-actin, FAE1 *and *Cruciferin*, accounted for over 95% of the transcript levels from the 32 genes (Table 2). The genes coding for storage proteins alone accounted for 87%, of which the *Napin *transcript was 77~78% of the total, *Oleosin *6~8%, and *Caleosin *and *Cruciferin *about 1~2%. For other non-storage protein genes, *β-CT*, *GAPDH*, *FAE1 *and *SAD *showed relatively higher transcript levels and accounted for 11% of the total transcripts (Table 2). The other 23 genes accounted for only about 3% of the total transcripts, of which *AAPT1*, *KAS3*, *MCAT*, *FatB*, *SUC1 *and *α-CT *had the lowest transcript levels (Table 2).

**Table 2:** Characterization of transcript profiles in developing seeds of *B. napus*.

Rank	Gene name	Mean transcript level (TL)		Ratio of Mean TL	Relative transcript level (RTL)(%)		Coefficient of variation (%)		Peak value		Day of peak(DAP)	
		ZS9	ZY821	ZS9/ZY821	ZS9	ZY821	ZS9	ZY821	ZS9	ZY821	ZS9	ZY821
1	*Napin*	46.92	50.22	0.93	76.89	75.41	156.10	140.02	178.46	172.97	35.00	45.00
2	*Oleosin*	3.91	5.47	0.71	6.40	8.21	133.12	167.69	13.50	23.93	40.00	40.00
3	*β-CT*	2.98	4.68	0.64	4.89	7.03	112.91	79.70	10.32	11.50	15.00	15.00
4	*Caleosin*	1.43	0.89	1.60	2.34	1.34	138.14	134.30	5.09	3.10	40.00	40.00
5	*FAE1*	1.09	0.60	1.80	1.78	0.91	103.81	110.91	3.30	2.06	30.00	40.00
6	*β-Actin*	1.00	1.00	1.00	1.64	1.50	40.57	46.89	1.69	1.56	45.00	40.00
7	*Cruciferin*	0.99	1.17	0.85	1.62	1.76	172.95	147.58	4.99	4.30	40.00	45.00
8	*SAD*	0.45	0.53	0.84	0.74	0.80	97.89	95.50	1.35	1.71	25.00	25.00
9	*GAPDH*	0.39	0.42	0.93	0.64	0.63	78.85	80.08	1.26	0.75	20.00	15.00
10	*GKTP*	0.38	0.23	1.66	0.62	0.34	138.40	38.09	1.64	0.38	45.00	45.00
11	*KCR2*	0.18	0.16	1.07	0.29	0.25	38.04	55.82	0.29	0.35	10.00	15.00
12	*FAD3*	0.17	0.08	2.09	0.28	0.12	79.09	61.19	0.38	0.15	25.00	30.00
13	*ACCase*	0.16	0.08	2.05	0.26	0.12	70.26	13.27	0.38	0.10	30.00	35.00
14	*FAD2*	0.16	0.14	1.09	0.26	0.22	65.42	80.85	0.35	0.42	25.00	25.00
15	*BcRK6*	0.15	0.22	0.65	0.24	0.34	122.44	103.15	0.44	0.60	15.00	20.00
16	*KAS1*	0.14	0.12	1.23	0.23	0.17	87.64	56.55	0.40	0.24	30.00	25.00
17	*BC*	0.10	0.09	1.13	0.17	0.14	46.83	47.12	0.17	0.14	10.00	10.00
18	*AGPase*	0.06	0.05	1.06	0.10	0.08	74.79	55.15	0.15	0.10	25.00	15.00
19	*KAS2*	0.05	0.04	1.13	0.08	0.06	74.76	57.42	0.12	0.08	10.00	10.00
20	*FAD6*	0.05	0.06	0.75	0.08	0.09	86.72	65.94	0.11	0.10	15.00	15.00
21	*PEPC*	0.04	0.03	1.39	0.06	0.04	61.29	62.14	0.07	0.05	10.00	15.00
22	*LPAAT*	0.04	0.03	1.22	0.06	0.04	54.31	55.81	0.06	0.05	25.00	15.00
23	*FatA*	0.03	0.05	0.68	0.05	0.07	65.09	71.64	0.07	0.10	15.00	10.00
24	*KACD*	0.03	0.03	1.24	0.05	0.04	64.80	50.96	0.06	0.04	10.00	10.00
25	*DGAT2*	0.03	0.04	0.86	0.05	0.05	47.42	50.37	0.06	0.06	15.00	15.00
26	*SUC1*	0.02	0.03	0.80	0.04	0.05	121.95	93.63	0.08	0.07	10.00	20.00
27	*α-CT*	0.02	0.03	0.78	0.04	0.04	50.17	57.06	0.04	0.05	20.00	20.00
28	*P*450	0.02	0.03	0.66	0.03	0.04	142.86	109.94	0.06	0.08	10.00	10.00
29	*FatB*	0.02	0.02	0.78	0.03	0.03	108.45	86.96	0.05	0.04	10.00	10.00
30	*KAS3*	0.01	0.03	0.49	0.02	0.05	119.26	109.93	0.05	0.09	15.00	20.00
31	*MCAT*	0.01	0.01	1.22	0.02	0.01	106.77	107.48	0.03	0.02	20.00	25.00
32	*AAPT1*	0.01	0.01	0.88	0.01	0.01	106.59	216.06	0.01	0.04	15.00	10.00

TL means mean transcript level, C.V. of TL means coefficient of variation of transcript level, and RTL means relative value of the transcript levels, DAP means days after pollination, or days between the pollination and the transcript peak occuring.

### Comparison of the Mean Transcript Levels between ZY821 and ZS9

For most of the genes investigated, the transcript levels changed with time during seed development (Fig. 2). Comparison of the mean transcript levels of ZS9 and ZY821 revealed that 11 of the 32 genes (*FAD3*, *ACCase, FAE1*, *GKTP*, *Caleosin, GAPDH*, *PEPC, KACD, KAS1, LPAAT *and *MCAT*) were upregulated by 20% to 109%, whereas 10 of the 32 genes (*KAS3, β-CT, BcRK6, P450, FatA, Oleosin, FAD6, FatB, α-CT *and *SUC1*) were downregulated by between 20% and 50% (Table 2 and Fig. 2).

Among the upregulated genes, *FAD3*, *ACCase *and *FAE1 *were the most remarkable. During 20–30 DAPs, the transcripts of *FAD3 *were 130~180% more abundant in ZS9 than in ZY821 (Fig. 2M). In ZY821 the *ACCase *transcript stayed stable at low levels, whereas in ZS9 it displayed high and low peaks at 25–30 DAPs and 40 DAP, about 4 fold and 2 fold the levels in ZY821, respectively (Fig. 2A). *FAE1 *gene transcripts were 150%–480% more abundant in ZS9 than in ZY821 during 15–35 DAPs. At 30 DAP, more than 4-fold higher *FAE1 *transcript levels were observed in ZS9 than in ZY821 (Fig. 2I). *Caleosin *and *GKTP *transcripts were more abundant only during the final stage of seed development (Fig. 2U, AC).

Among the downregulated genes, *KAS3 *showed significantly lower expression at 10 and 20 DAPs (Fig. 2H), *FatA *at 10–20 DAPs (Fig. 2O), *BcRK6 *and *P450 *at 20 DAP (Fig. 2AD, Y), *β-CT *at 20–40 DAPs (Fig. 2D) and *Oleosin *at 40–45 DAPs (Fig. 2T).

### Comparison of the ZY821 and ZS9 Transcript Peak Levels

The value and time of a transcript's peak levels are an indication of gene activity and regulation. ZY821 and ZS9 had different peak values and times for the expression of many of the genes investigated. The peak value was 338%, 288% and 162% higher in ZS9 than in ZY821 for *FAD3*, *ACCase *and *GKTP*, respectively (Table 2 and Fig. 2M, A, AC), and 40–60% higher in ZS9 than in ZY821 for *KAS2*, *MCAT*, *AGPase*, *PEPC*, *KACD*, *FAE1, Caleosin*, *KAS1 *and *GAPDH *(Table 2 and Fig. 2G, E, X, Z, J, I, U, F, AE). The peak value was over 60% lower for *AAPT1 *and 20–40% lower for *Oleosin*, *KAS3*, *FatA*, *BcRK6*, *SAD *and *P450 *in ZS9 than in ZY821 (Fig. 2S, T, H, O, AD, K, Y).

According to the peak time of gene expression, the 32 genes can be clustered into 4 groups. In ZS9, 12 genes (*P450, KCR2, SUC1, FatB, BC, KAS2, PEPC, KACD, AAPT1, FatA, BcRK6, KAS3*) peaked at 10 or 15 DAPs, 8 genes (*α-CT, MCAT, GAPDH, SAD, FAD2, LPAAT, AGPase, FAD3*) at 20–25 DAPs, 4 genes (*FAE1, KAS1, ACCase *and *Napin*) at 30–35 DAPs and 4 genes (*Oleosin, Cruciferin, Caleosin*, and *GKTP*) at 40–45 DAPs. In ZY821, 15 genes (*P450, FatB, BC, KAS2, KACD, AAPT1, FatA, KCR2, PEPC, β-CT, DGAT2, FAD6, GAPDH, LPAAT *and *AGPase*) peaked at 10 or 15 DAPs, 8 genes (*SUC1, KAS3, BcRK6, α-CT, MCAT, SAD, FAD2, KAS1*) at 20–25 DAPs, 2 genes (*ACCase *and *FAD3*) at 20–25 DAPs, and 7 genes (*FAE1, Oleosin, Caleosin, β-actin, Napin, Cruciferin *and *GKTP*) at 40–45 DAPs. In most cases, the difference in peak times between the two cultivars was no more than 5 days, but *LPAAT *and *AGPase *peaked 10 days later in ZS9 than in ZY821, whereas *SUC1 *and *Napin *peaked 10 days earlier.

### Comparison of Gene Expression Patterns between ZY821 and ZS9

Different genes showed different degrees of variation in their transcript levels over the course of seed development, as shown by the variation coefficients in Table 2. In ZY821, *AAPT1*, *Oleosin*, *Cruciferin*, *NapinB*, *Caleosin*, *FAE1*, *P450 *and *KAS3 *had relatively higher variation, whereas in ZS9, *Cruciferin, NapinB, P450, GKTP, Caleosin, Oleosin, BcRK6 *and *SUC1 *did. *β-actin *had the lowest variation in both cultivars, serving as a satisfactory control gene in this study.

The 32 genes can be divided into five groups according to their expression patterns.

The first group is characterized by a basic transcript level at the initiation of seed formation, a rapid rise to a peak at 35–40 DAP, and then by a gradual decline. The genes for storage proteins, such as *Napin*, *Caleosin*, *Cruciferin*, and *Oleosin*, possessed this pattern (Fig. 2T, U, V, W). The expression patterns of these genes were much like the accumulation pattern of the total fatty acids, C18:1 in ZS9 (Fig. 1D) and C22:1 in ZY821 (Fig. 1J).

The second group is characterized by a bell-shape transcript curve, with a moderate level of expression at the initial stage followed by a gradual increase in transcripts at the middle stage and a decline to the initial level at the final stage. The genes *SAD*, *FAD2*, *FAD3*, *KAS1*, *AGPase*, *MCAT *and *FAE1 *possessed this pattern (Fig. 2K, L, M, F, X, E, I). These enzymes may function in fatty acid synthesis. The accumulation of the fatty acids C18:1 and C20:1 in ZY821 (Fig. 1D, H) also fell into such a pattern.

The third group is characterized by a low transcript level at the beginning, followed by further decreasing expression throughout seed development, as exhibited by *FAD6*, *FatA*, *FatB*, *α-CT*, *BC*, *PEPC*, *KCR2*, and *KACD *(Fig. 2N, O, P, C, B, Z, J). The fatty acids C16:0, C18:0, C18:2, C20:0, and C22:0, which are minor components in mature seeds once synthesized in early seed development, also showed such a pattern (Fig. 1B, C, E, G, I).

The fourth group is characterized by a stable expression or a gently rise and fall in expression pattern. *GKTP, ACCase, KAS2, DGAT2 *and *β-actin *are included in this group (Fig. 2AC, A, G, R). The fatty acid α-C18:3 had a similar pattern to this group (Fig. 1F). The fifth group is characterized by a high level of expression at the initiation of seed development, peaking at 10 or 15 DAP, followed by an abrupt decline, as exhibited by *β-CT*, *BcRK6*, *KAS3*, *AAPT1*, *SUC1 *and *P450 *(Fig. 2D, AD, H, S, AA).

A comparison of ZS9 and ZY821 indicated that most of the genes displayed similar expression patterns, although a few of the genes, such as *ACCase*, *FAE1 *and *GKTP*, had obvious differences between ZY821 and ZS9 (Fig. 2A, I, AC). *ACCase *had a bell-shape expression pattern in ZS9, putting it in the second group, whereas it had a flat pattern in ZY821, putting it in the third group (Fig. 2A). *FAE1 *was in the first group of expression pattern in ZY821 but had a bell-shape pattern in ZS9, putting it in the second group (Fig. 2I). *GKTP *also showed a significant difference, with a significant rise in transcript levels at the mature stage of seed development in ZS9, placing it in third group, although it had a very low level throughout all stages of seed development in ZY821 (Fig. 2AC).

### Correlation between fatty acid accumulation and gene expression

Correlation analysis was performed between transcript profiles of the genes and accumulation patterns of the fatty acids. The results showed that the two cultivars had different correlation patterns (Table 3). For the Low Erucic Acid Rapeseed (LEAR) ZS9, the total amount of fatty acids was highly correlated with *Caleosin *and correlated with *Cruciferin *and *Oleosin*; the amount of C16:0 was highly correlated with *BC, PEPC *and *FAD6 *and correlated with *Oleosin, α-CT, KAS2, β-CT, BcRK6, SUC1, DGAT2, FatA, P450, FatB, AAPT1*, and *KCR2*; C18:0 was highly correlated with *SUC1*, *β-CT*, *BC, PEPC, FAD6, FatA, P450, FatB*, and *AAPT1*, positively correlated with *KCR2, DGAT2, KAS3, BcRK6, KAS2*, and *α-CT*, and negatively correlated with *Oleosin*; C18:1 was highly negatively correlated with *BC, FAD6 *and *PEPC*, negatively correlated with *AAPT1, FatB, SUC1, P450, β-CT, FatA, KCR2, BcRK6, KAS3, DGAT2, α-CT*, and *KAS2 *and positively correlated with *Caleosin *and *Oleosin*; C18:2 was highly positively correlated with *BC, FatB, AAPT1, FAD6, SUC1, PEPC, P450 *and *KCR2 *and correlated with *β-CT, FatA, BcRK6, KAS3, DGAT2 *and *α-CT*; C18:3 was highly positively correlated with *ACCase *and correlated with *FAD3*. For High Erucic Acid Rapeseed (HEAR) ZY821, the total amount of fatty acids was highly negatively correlated with *KCR2*, negatively correlated with *ACCase *and positively correlated with *FAE1*. C16:0 was highly positively correlated with *KCR2*, positively correlated with *AGPase, PEPC, FAD6, ACCase, FatA, GKTP, LPAAT*, and *FatB *and negatively correlated with *FAE1*; no significant correlation for C18:1 was found with any gene; C18:2 was highly positively correlated with *KCR2 *and highly negatively correlated with *FAE1*, positively correlated with *FatB, ACCase*, and *PEPC *and negatively correlated with *Caleosin*; C18:3 was correlated only with *FAD3*; C20:0 was highly positively correlated with *KCR2 *and correlated with *AGPase, ACCase*, and *GKTP*; C20:1 was highly positively correlated with *FAE1*, highly negatively correlated with *KCR2 *and negatively correlated with *PEPC*; C22:1 was highly positively correlated with *Caleosin *and *Cruciferin*, highly negatively correlated with *KAS1, BC, KCR2 *and *β-CT*, positively correlated with *Napin, Oleosin*, and *FAE1*, and negatively correlated with *KACD, FAD6, FatA, FatB*, and *LPAAT*.

**Table 3:** The correlation coefficients for the gene expression pattern and the fatty acid accumulation pattern1),2).

Note^3)^		Total-ZS9	C16:0-ZS9	C18:0-ZS9	C18:1-ZS9	C18:2-ZS9	C18:3-ZS9	Total-ZY821	C16:0-ZY821	C18:0-ZY821	C18:1-ZY821	C18:2-ZY821	C18:3-ZY821	C20:0-ZY821	C20:1-ZY821	C22:0-ZY821	C22:1-ZY821
2	*Oleosin*	**0.83**	**-0.80**	**-0.74**	**0.76**	-0.62	-0.22	0.57	-0.63	-0.58	-0.03	-0.63	-0.62	-0.49	0.44	-0.41	**0.81**
2	*Cruciferin*	**0.81**	-0.65	-0.58	0.61	-0.49	-0.23	0.48	-0.62	-0.58	-0.10	-0.63	-0.45	-0.56	0.42	-0.38	**0.83**
2	*Caleosin*	**0.85**	**-0.77**	-0.70	**0.73**	-0.60	-0.33	0.48	-0.68	-0.62	-0.17	-0.72	-0.30	-0.50	0.54	-0.37	**0.90**
2	*β-CT*	-0.61	**0.82**	**0.84**	**-0.82**	**0.81**	-0.12	-0.51	0.51	0.44	0.40	0.55	0.48	0.21	-0.41	0.16	**-0.84**
2	*FAD3*	-0.34	0.33	0.22	-0.26	0.11	**0.88**	-0.41	0.33	0.34	0.18	0.41	**0.81**	0.15	-0.25	0.22	-0.62
2	*BC*	-0.68	**0.92**	**0.96**	**-0.94**	**0.95**	-0.10	-0.56	0.62	0.60	0.16	0.69	0.54	0.40	-0.53	0.39	**-0.87**
2	*PEPC*	-0.53	**0.85**	**0.90**	**-0.85**	**0.86**	-0.17	-0.70	**0.77**	0.71	-0.21	**0.71**	-0.48	0.60	**-0.80**	0.54	-0.59
2	*FAD6*	-0.60	**0.88**	**0.91**	**-0.87**	**0.86**	-0.10	-0.58	**0.74**	0.69	-0.13	0.69	0.22	0.55	-0.58	0.55	**-0.77**
2	*ACCase*	0.02	0.03	-0.05	0.04	-0.17	**0.91**	-0.71	**0.79**	**0.72**	-0.24	**0.71**	-0.02	**0.71**	-0.67	0.57	-0.69
2	*FatA*	-0.51	**0.81**	**0.86**	**-0.80**	**0.80**	-0.08	-0.70	**0.72**	0.70	-0.11	0.70	0.26	0.47	-0.64	0.57	**-0.76**
2	*FatB*	-0.51	**0.82**	**0.89**	**-0.83**	**0.87**	-0.26	-0.58	**0.78**	**0.76**	-0.32	**0.74**	0.20	0.64	-0.62	0.69	**-0.72**
2	*AAPT1*	-0.52	**0.82**	**0.89**	**-0.83**	**0.86**	-0.20	-0.51	0.59	0.67	-0.55	0.62	0.29	0.57	-0.55	**0.78**	-0.43
2	*KCR2*	-0.48	**0.77**	**0.82**	**-0.80**	**0.86**	-0.06	**-0.84**	**0.95**	**0.95**	-0.39	**0.94**	0.14	**0.90**	**-0.86**	**0.82**	**-0.85**
1	*FAE1*	0.49	-0.57	-0.61	0.58	-0.61	0.58	**0.75**	**-0.88**	**-0.84**	0.21	**-0.87**	0.18	-0.63	**0.89**	-0.67	**0.78**
1	*Napin*	0.43	-0.51	-0.49	0.45	-0.32	0.06	0.59	-0.65	-0.61	-0.02	-0.64	-0.48	-0.59	0.45	-0.43	**0.81**
1	*α-CT*	-0.46	**0.76**	**0.80**	**-0.74**	**0.74**	-0.14	0.03	0.12	0.08	0.07	0.08	0.47	-0.02	0.08	0.07	-0.27
1	*KAS1*	-0.29	0.35	0.35	-0.34	0.26	0.11	-0.59	0.55	0.48	0.51	0.63	0.18	0.27	-0.56	0.10	**-0.89**
1	*KAS2*	-0.50	**0.75**	**0.73**	**-0.72**	0.69	0.31	-0.43	0.40	0.30	0.18	0.30	0.02	0.13	-0.31	0.11	-0.48
1	*β-actin*	0.38	-0.40	-0.48	0.46	-0.56	**0.77**	-0.07	-0.13	-0.08	-0.45	-0.20	0.54	0.06	0.23	0.15	0.29
1	*BcRK6*	-0.46	**0.79**	**0.85**	**-0.79**	**0.80**	-0.19	-0.28	0.35	0.32	0.03	0.32	0.36	0.12	-0.22	0.25	-0.46
1	*KAS3*	-0.47	**0.78**	**0.82**	**-0.76**	**0.76**	-0.16	-0.45	0.47	0.44	-0.01	0.44	0.27	0.21	-0.39	0.36	-0.54
1	*KACD*	-0.43	0.50	0.43	-0.44	0.31	0.70	-0.55	0.58	0.56	0.16	0.63	0.51	0.32	-0.50	0.37	**-0.81**
1	*SUC1*	-0.50	**0.82**	**0.89**	**-0.83**	**0.86**	-0.23	-0.42	0.46	0.45	-0.08	0.44	0.40	0.24	-0.35	0.40	-0.53
1	*AGPase*	-0.52	0.54	0.43	-0.46	0.30	0.69	-0.56	**0.72**	0.66	-0.18	0.68	-0.27	0.72	-0.65	0.49	-0.63
1	*DGAT2*	-0.46	**0.78**	**0.82**	**-0.76**	**0.75**	-0.10	-0.59	0.65	0.62	-0.12	0.62	0.23	0.40	-0.56	0.53	-0.67
1	*GKTP*	0.32	-0.20	-0.08	0.19	-0.08	-0.69	-0.57	**0.75**	**0.71**	-0.38	0.69	-0.37	**0.74**	-0.70	0.61	-0.53
1	*LPAAT*	-0.38	0.66	0.67	-0.61	0.55	0.04	-0.57	**0.75**	**0.72**	-0.28	0.70	0.25	0.60	-0.58	0.65	**-0.71**
1	*P*450	-0.51	**0.82**	**0.88**	**-0.83**	**0.86**	-0.22	-0.47	0.61	0.64	-0.38	0.61	0.40	0.50	-0.48	0.68	-0.56
0	*SAD*	-0.36	0.14	0.00	-0.10	-0.04	0.55	-0.04	-0.21	-0.23	0.59	-0.11	0.34	-0.29	0.13	-0.44	-0.13
0	*MCAT*	-0.47	0.13	0.03	-0.13	0.04	0.07	-0.47	0.54	0.48	0.09	0.55	-0.29	0.52	-0.55	0.24	-0.57
0	*FAD2*	-0.17	-0.06	-0.19	0.08	-0.13	0.57	-0.44	0.51	0.47	0.07	0.54	-0.19	0.51	-0.51	0.24	-0.55
0	*GAPDH*	-0.46	0.14	0.03	-0.14	0.04	0.20	-0.35	0.48	0.43	-0.14	0.45	-0.39	0.54	-0.46	0.29	-0.36

Note: 1) Significant levels: r0.05 = 0.707, r0.01 = 0.834

2) Bold numbers indicate the correlation is significant (0.05 probability level); Underlined bold numbers indicate the correlation is highly significant (0.01 probability level).

3) Zero means the gene shows no significant correlation with any fatty acid in either cultivar. One means the gene shows no significant correlation with any fatty acid in one cultivar; two means the gene shows significant correlations in both cultivars.

The genes *Napin, KACD, AGPase, GKTP*, and *LPAAT *in LEAR ZS9 and the genes *α-CT, KAS2, BcRK6, KAS3, SUC1, DGAT2*, and *P450 *in HEAR ZY821 showed no significant correlation with any fatty acids or with the total amount of fatty acids.

## Discussion

Many efforts by plant breeders have focused on strategies to enhance both the quantity and the quality of seed storage reserves and, more recently, molecular genetics approaches have been used to modify seed components. The biochemical pathways that produce these different storage components are largely known [7-9], but the factors that determine the relative proportions of the different storage components are not well understood. This study has provided a new data set describing the patterns of expression of 32 rapeseed genes during storage product accumulation in seed development and characterizing the response of gene expression to quality improvement in rapeseed.

The present study indicated that the expression levels of specific genes changed with the selection pressures when ZS9 and ZY821 were compared. Seven of the 32 genes (*FAD3*, *ACCase*, *FAE1*, *GKTP*, *Caleosin*, *GAPDH*, and *PEPC*) were upregulated by 30% to 109%, whereas 5 genes (*KAS3*, *β-CT*, *BcRK6*, *P450 *and *FatA*) were down-regulated 50%–30% in ZS9 compared with ZY821. These changes may come from selection pressures or from genetic drift during breeding.

### Genes studied

Lipid synthesis depends on the correct spatial and temporal activity of many gene products [7,8]. These genes execute their function in three stages: fatty acid synthesis in the plastid, triacylglycerol (TAG) synthesis in the endoplasmic reticulum (ER) and assembly into an oil body [10].

The first fatty acid synthesis stage commences with *ACCase*, catalyzing the carboxylation of acetyl-CoA to malonyl-CoA [11]. Most plants have two forms of *ACCase*, the homomeric form in the cytosol, composed of a single large polypeptide catalyzing the individual carboxylation reactions [12], and the heteromeric form in plastids, composed of four subunits, including Biotin Carboxylase (*BC*) [13], Biotin Carboxyl Carrier Protein (*BCCP*) [14], α-Carboxyltransferase (*α-CT*) [15] and β-Carboxyltransferase (*β-CT*) [16].

The next enzyme is *MCAT *(Malonyl-CoA-acyl carrier protein transacylase), which catalyzes the transfer of malonyl-CoA to the holo acyl carrier protein (ACP), generating malonyl-ACP [17]. Beta-ketoacyl-acyl carrier protein synthase III (*KAS3*) catalyzes the subsequent condensation and transacylation of acetyl-CoA with malonyl-ACP [18]. *KAS1 *catalyzes the subsequent condensation from C4- to C14-ACP in the plastid [19], and *KAS2 *elongates 16:0-ACP to 18:0-ACP in the plastid [20].

The synthesized 18:0-ACP is converted to oleic acid (18:1) by *SAD *[21]. In HEAR cultivars such as ZY821, the oleic acids (18:1) continue the elongation process to synthesize C20- to C22- fatty acids such as erucic acid (22:1), which involves four enzymatic steps: condensation by 3-ketoacyl-CoA synthase (*FAE1*) [22], reduction by 3-ketoacyl-CoA reductase (*KCR*) [23], dehydration by 3-hydroxyacyl-CoA dehydratase (*KACD*) [24] and finally reduction by trans-2,3-enoyl-CoA reductase. In LEAR cultivars, such as ZS9, fatty acids do not elongate any more due to the disfunction of *FAE1 *[25,26].

Oleic acid (18:1) can be further desaturated to oleate acids (18:2) by *FAD2 *[27] and *FAD6 *[28] in the cytosol and the plastid, respectively. Cytosolic and plastid ω-3 desaturations that result in the production of linolenic acids (18:3) are controlled by *FAD3 *[29] and *FAD7 *[30], respectively.

The final step in the plastid is the release of fatty acids from ACP by thioesterase, resulting in the formation of dissociated fatty acids [31]. Two distinct but related thioesterase gene classes exist in higher plants. *FatA *is an acyl specificity 18:1>>18:0>>16:0 thioesterase [32]. In contrast, *FatB*, encoding thioesterases, has a specificity for 16:0>18:1>18:0 fatty acids [33]. Fatty acids are exported to the cytosol and activated to their acyl-CoA derivatives.

In the following stage, fatty acids are incorporated into glycerolipids through the acylation of glycerol-3-phosphate by acyltransferases, producing TAG. *De novo *phosphatidic acid biosynthesis in plants occurs in three steps: first, the acylation of the sn-1 position of glycerol-3-phosphate gives rise to lysophosphatidic acid, which is catalyzed by Glycerol-Phosphate Acyltransferase (*GPAT*) [34]; second, the acylation of the sn-2 position forms phosphatidic acid, which is catalyzed by a lysophosphatidic acid acyltransferase (*LPAAT*) [35]. Diacylglycerol acyltransferase (*DGAT*) catalyzes the final step of the TAG synthesis pathway [36].

The TAG assembles with oil body proteins to form oil bodies. There are four predominant seed storage proteins: *Cruciferin*, *Caleosin*, *Oleosin *and *Napin*. *Cruciferin *is a high molecular weight neutral complex of 12S proteins, composed of six subunit pairs [37]. *Napin *is a low molecular weight basic 1.7S protein, composed of two disulfide-linked polypeptide chains [38]. *Caleosin *is a Ca^2+^-binding oil-body surface protein [39] playing a role in both the normal modification of storage vacuole membranes and the interaction of oil bodies with vacuoles [40]. *Oleosins *are oil body-associated proteins that cover and stabilize oil bodies during TAG accumulation [41].

### Relative transcript levels of genes in the developing seed

According to DeLisle and Crouch [42], mRNA is about 1% of the total RNA in *B. napus *embryos. *Cruciferin *and *Napin *represent 11 and 8%, respectively, of embryonic mRNA based on mass [42]. *Cruciferin *and *Napin *constitute 20% and 60% of the total protein content of mature seeds, respectively [43]. Moreover, *Napin *(12.5–14.5 kDa) is about 25 fold smaller in mass (2S albumin) than *Cruciferin *(12S globulin, 300–310 kDa) [43]. All of this implies that *Napin *mRNA is the most abundant and is much higher in copy number than *Cruciferin *mRNA during rapeseed development, in agreement with our results. With the strongest expression in seed, the *Napin *promoter has been popularly used for genetic engineering of seed components.

No estimate of the relative mRNA abundance has been reported for *Oleosin *and *Caleosin *in *B. napus *thus far, but it has been reported that *Oleosins *are low molecular weight (15–26 kDa) alkaline proteins that represent about 2–8% of the total seed proteins [44], and *Caleosins *are present at relatively low levels and are mainly bound to microsomal membrane fractions [45]. Our data are the first on the relative abundance of *Oleosin *and *Caleosin *transcripts in rapeseed development, indicating that *Oleosin *transcripts are the second most abundant in copy number after *Napin *among the 4 storage proteins (Table 2).

No significant difference was found in the relative abundance of seed mRNA between ZY821 and ZS9, indicating that the genes are expressed in a highly organized and coordinated way and in a stable molar amount during seed development.

### Upregulated genes (*FAD3*, *ACCase*, *FAE1*, *GKTP*, *Caleosin*, *GAPDH*, *PEPC*)

*PEPC*, upregulated by 39% in ZS9 compared with ZY821, is an enzyme in the family of carboxylyases that catalyzes the addition of CO_2 _to phosphoenolpyruvate (PEP) to form the four-carbon compound oxaloacetate. Seeds of transgenic bean plants expressing *Corynebacterium glutamicum PEPC *in a seed-specific manner had a more rapid shift in metabolic fluxes from sugars/starch into organic acids and free amino acids [46], resulting in an accumulation of up to 20% more protein per gram dry seed weight in the transgenic seeds and a 20–30% higher seed dry weight [46]. We show that *PEPC *upregulation may result in increased C16:0, C18:0 and C18: 2 but decreased C18:1 (Table 3).

*ACCase*, upregulated by 105% in ZS9 compared with ZY821, is the homomeric ACCase that catalyzes the first committed step of fatty acid synthesis, the carboxylation of acetyl-CoA to malonyl-CoA. Targeting a homomeric *ACCase *to rapeseed plastids under the control of a seed-specific promoter resulted in higher *ACCase *activity and increased oil yield by 3–5% on a dry seed weight basis [12]. Over-expression of *ACCase *in the amyloplasts of potato tubers led to an increase in fatty acid synthesis and a more than 5-fold increase in the amount of TAG [47]. *ACCase *is a key target to increase seed oil by genetic engineering [12,47]. In the present study, increased *ACCase *activity was accompanied with increased C18:3 in ZS9 and with increased C16:0, C18:0, C20:2 and C18:2 and decreased total fatty acids in ZY821. Considering that C18:3 does not change much with seed development, increased C18:3 must be consumed at a similar amount during seed development.

*FAE1*, upregulated by 80% in ZS9 compared with ZY821, is a key gene responsible for fatty acid elongation from 18:1 to 22:1 or erucic acid biosynthesis in *Brassica*. Seed-specific expression in *Arabidopsis thaliana *resulted in up to a 12-fold increase in the proportion of erucic acid. On the other hand, in transgenic high-erucic *Brassica carinata *plants, the proportion of erucic acid was as high as 51.9% in the best transgenic line, a net increase of 40% compared with wild type [48]. *FAE1 *mRNA was normally transcribed in both HEAR and LEAR cultivars but a 57 kDa protein was found only in HEAR seeds, whereas it was absent in all LEAR cultivars using an antibody against *FAE1 *[26,49]. It was beyond our expectation that the *FAE1 *transcript was more abundant in LEAR ZS9 than in HEAR ZY821 (Fig. 2I). The upregulated *FAE1 *transcript observed in this study (Fig. 2I) might be due to synthesized *FAE1 *mRNA that was not used for the blocked synthesis of functional *FAE1 *enzyme.

*FAD3 *was upregulated by 109% in ZS9 compared with ZY821 (Table 2) and was significantly correlated only with C18:3 in the present study (Table 3). No significant difference in C18:3 content is found during seed development between ZY821 and ZS9 (Fig. 1D). Why the *FAD3 *transcript level was up-regulated by so much and no more C18:3 was found in ZS9 needs further investigation. It is worthwhile to notice that the *FAD3 *and *FAE1 *changed coodinately (Fig. 2I, M), consistent with previous reports that they are both regulated by abscisic acid (ABA) [5]. Some key transcription factors involved in lipid metabolism are under the influence of ABA signaling [5]. The upregulation of *FAD3 *found in this study may be linked through some regulation cofactors with the upregulation of *FAE1 *in ZS9.

Abscisic acid induces *Oleosin *expression [50], and *FAD3 *and *FAE1 *also are regulated by abscisic acid [51-53]. The upregulation of *FAD3*, *FAE1 *and *Oleosin *found in this study might be owing to the same regulation cofactor. The breeding pressure may have selected for the action of this cofactor.

*Caleosin*, one of the storage proteins, was upregulated by 60% in ZS9 compared with ZY821 (Table 2). It was significantly correlated with total fatty acids and C18:1 in ZS9 and with C22:1 in ZY821 (Table 3). Meanwhile, transcrips of other storage proteins (*Cruciferin*, *Oleosin *and *Napin*) were all downregulated. Unlike *Oleosin*, *Caleosin *may be involved in signal transduction via calcium binding or phosphorylation/dephosphorylation in processes such as membrane trafficking and lipid-body fusion [39]. During germination *Caleosin *plays a role in the degradation of storage lipid in oil bodies [40]. Upregulation of *Caleosin *implies enhanced oil deposition and improved seed germination in ZS9.

*GKTP *(synonym *KAT2*), upregulated by 66% in ZS9 compared with ZY821, codes for the precursor of glyoxysomal 3-ketoacyl-CoA thiolase, the last enzyme in the beta-oxidation of fatty acids in plant glyoxysomes. Expression of this enzyme is required for the timely onset of natural and dark-induced leaf senescence in *Arabidopsis *[54]. In this study, the transcript abundance of *GKTP *is correlated with C16:0, C18:0 and C20:0 in ZY821 but not with any fatty acid in ZS9. The reason for this difference is not clear.

*GAPDH*, upregulated by 56% in ZS9 compared with ZY821, encodes glyceraldehyde 3-phosphate dehydrogenase, which catalyzes the sixth step of glycolysis and thus serves to break down glucose for energy and carbon molecules. In addition to this long established metabolic function, *GAPDH *has recently been implicated in several non-metabolic processes, including transcription activation, initiation of apoptosis, and ER to Golgi vesicle shuttling. In this study, *GAPDH *showed relatively stable and high expression among the non-storage protein genes during seed development. The activity of *GAPDH *was not found to be correlated with any fatty acid biosynthesis in this study (Table 3).

### Down-regulated genes (*KAS3*, *β-CT*, *BcRK6*, *P450 *and *FatA*)

*KAS3 *was down regulated by 51% in ZS9 compared with ZY821 (Table 2). It was also found to be significantly correlated with C16:0, C18:0 and C18:2 in ZS9 but not in ZY821. Katayoon *et al*. indicated that overexpression of *KAS3 *in tobacco plants reduces the rate of lipid synthesis and increases the levels of C16:0 in leaves. The transgenic *B. napus *seeds overexpressing *KAS3 *driven by *Napin *also contained lower levels of oil compared with wild-type [55]. In addition, the rate of lipid synthesis in transgenic rapeseed seeds was notably slower than that of the wild-type seeds [55]. Therefore, down-regulation of *KAS3 *might result in reduced saturated fatty acids and increased C18:2 in ZS9 (Fig. 1B, C, G). The same situation is also found for *P450*, *FatA*, *FatB*, *AAPT1*, *KCR2*, *α-CT*, *β-CT, BC*, *SUC1, BcRK6 *and *KAS2 *in this study (Table 3).

*FatA *was downregulated 32% in ZS9 compared with ZY821 (Table 2). It was shown in this study that transcript levels of *FatA *were negatively correlated with C18:1 in ZS9 and with C22:1 in ZY821 (Table 3). In both cultivars, the transcripts of *FatA *peaked at 10 or 15 DAPs, and positively correlated with C16:0 and C18:0 (Table 3). Thioesterases *FatA *and *FatB *play an essential role in the partitioning of de novo-synthesized fatty acids between the prokaryotic and eukaryotic pathways. *FatA *determines the in vivo levels of C18:1 that move out from the plastid [31]. Downregulated *FatA *means lower levels of C18:1 move out from the plastid. This is reasonable because C18:1 was not consumed for C22:1 in ZS9, high accumulation of C18:1 must inhibit transcription of *FatA*. Similar situations were found for *FatB *(Table 3).

As one of the 4 subunits of the multisubunit plastidial *ACCase*, *β-CT *was downregulated 36% in ZS9 compared with ZY821. The transcript abundance of *β-CT *was much higher than other subunits of the multisubunit plastidial *ACCase *and ranked the third just next to *Napin *and *Oleosin *in both cultivars (Table 2). This is probabally because *β-CT *gene is the only component of plant lipid metabolism known to be encoded by the plastid genome [11]. We found downregulation of *β-CT *was accompanied by increased C18:1 in ZS9 and higher C22:1 in ZY821 (Table 3), while reduction of the expression of *β-CT *was reported to result in reduced fatty acid synthesis and even damage of embryo development [56].

*BcRK6*, downregulated 36% in ZS9 compared with ZY821, is a receptor kinase, catalyzing the key step in steroid perception and signaling in plants [57]. This enzyme was active at 10–20 DAP and was reduced sharply afterward. Decreased *BcRK6 *transcripts were significantly accompanied by increased C18:1 accumulation in ZS9 (Table 3). The reason for the downregulation of *BcRK6 *in ZS9 observed at 20 DAP needs further investigation.

Cytochrome *P450 *catalyzes a monooxygenase reaction, e.g., insertion of one atom of oxygen into an organic substrate (RH), whereas the other oxygen atom is reduced to water using a plethora of both exogenous and endogenous compounds as substrates for enzymatic reactions, producing a wide cultivar of susceptibility to specific toxins. Cytochrome *P450 *enzymes are present in most other tissues of the body and play important roles in hormone synthesis and breakdown (including estrogen and testosterone synthesis and metabolism), cholesterol synthesis, and vitamin D metabolism. This gene showed significant correlations in ZS9 but correlations were not found in ZY821. The reasons for this difference are not clear.

### Correlation analysis

Most of the genes investigated in this study showed significant correlation with fatty acid accumulation (Table 3). We found that the correlation of the genes in ZS9 was significantly different from that in ZY821. For example, significant correlation was not found with any fatty acid in ZS9 but it was found with 6 fatty acids in ZY821; *ACCase *is highly significantly correlated with C18:3 in the LEAR ZS9 but not in ZY821, whereas in ZY821, *ACCase *showed significant correlation with many other fatty acids, including total fatty acids C16:0, C18:0, C20:0, and C18:2. Exceptionally, in both cultivars *FAD3 *showed significant correlation only with C18:3, in accordance with its defined function for the production of C18:3 from C18:2 [27].

In ZS9, more tighter correlations were found between gene transcript levels and fatty acid accumulation patterns. In ZS9, thirteen genes including *P450, FatB, AAPT1, KCR2, DGAT2, PEPC, FAD6, FatA, BC, SUC1, Oleosin, α-CT*, and *β-CT*, whereas only four genes in ZY821, i.e., *KCR2, FAE1, ACCase *and *FatB*, showed significant correlation with at least four fatty acids, indicating that they play important parts in the storage accumulation of seed development. However, in both cultivars, the genes *SAD, MCAT *and *FAD2*, which among the down regulated genes showed no significant correlation with either any fatty acids or the total amount of fatty acids, did not respond to the breeding progress for fatty acid composition in rapeseed.

According to the frequency of significant correlations, *KCR2 *seemed of the highest importance in the fatty acid accumulation process. Higher *KCR2 *activity is associated with higher C16:0, C18:0, and C18:2 in both cultivars, lower C22:1 and total fatty acids in ZY821, and lower C18:1 in ZS9. *Bn-KCR *was preferentially expressed in seeds and roots. The co-expression of *Bn-FAE1 *and *Bn-KCR *observed in the HEAR cultivar during seed development was different from that of the LEAR cultivar, suggesting that the expression of both genes was directly or indirectly linked [23]. To our knowledge, this is the first time a close correlation between *KCR2 *and fatty acid accumulation has been shown.

## Conclusion

This paper illustrated the breeding response of the transcription levels of 32 genes in developing rapeseed seeds of two cultivars. Both cultivars showed similar transcription profiles, with the *Napin *gene transcribed most predominantly. Selective pressure toward zero erucic acid, low glucosinolate, high oleic acid and high oil content as well as high yield resulted in both higher *FAE1*, *FAD3*, *Caleosin*, *LPAAT*, *KACD*, and *ACCase *and lower *FatB*, *FAD2*, *SUC1*, *BcRK6*, *KAS3*, and *MCAT *expression and in altered correlations between these genes during the storage accumulation of seed development. These results provide insight into the structure of the primary transcriptional networks that coordinate the metabolic responses to seed developmental programs in rapeseed.

## Methods

### Plant Material

Both ZY821 and ZS9 are conventional rapeseed cultivars (*B. napus*), bred by our Institute. ZS9 was the offspring of ZY821 by crossing. Because they are two Chinese representative rapeseed cultivars bred during the past 20 years, they show some major differences, as described in Table 1.

During the flowering stage, flower buds were self-pollinated and bagged after removal of flowered and young buds in the main inflorescence. Developing pods were harvested at 5-day intervals, 10 to 45 DAP. Dissected seeds were frozen immediately in liquid nitrogen and stored at -70°C until gas chromatographic (GC) analysis or RNA extraction.

### Selection of Genes

Thirty-two genes involved in the biosynthesis of storage products in rapeseed (Table 4) were identified by exhaustive database searches and by referring to the comprehensive lipid gene catalog provided by Mekhedov *et al. *[7]. The selected genes cover all of the major biochemical events in the biosynthesis of storage products in rapeseed [7,58]. Seed oil is synthesized in three stages: *de novo *fatty acid synthesis from C2 to C16 in the plastid, fatty acid elongation from C16 to C22, fatty acid desaturation and TAG synthesis in the ER and assembly into the oil body [10]. The following were identified: four eukaryotic *ACCase *and prokaryotic plastidial *ACCase *subunit genes, which catalyze the first reaction in the fatty acid biosynthetic pathway, seven fatty acid elongase genes, which catalyze fatty acid elongation from varied lengths of carbon chains, four fatty acid desaturase genes, which catalyze the subsequent desaturation of the lipids to highly unsaturated forms, two thioesterase genes, which release free fatty acid from an acyl-ACP, three glycerolipid synthesis genes, which catalyze the acylation reactions of free fatty acids with glycerol-3 phosphate, four storage protein biosynthesis genes, six basic metabolism genes, and two housekeeping genes (Table 4). The functions and roles of these genes have been explained in detail by Mekhedov *et al*. and Ohlrogge and Browse [7,58].

**Table 4:** Primer pairs and amplicon sizes of the genes analyzed in this study.

Categry	Accession number	Gene name	Gene annotation	Primer sequence	Amplicon size(bp)	PCR efficency
ACCase	X77382	*ACCase*	Homomeric acetyl CoA carboxylase	F:5'AGGACTTGCCAATCTTCTAAAC3'	157	0.973
				R:5'AGCTTCTTTCACCGTAGGACAC3'		
	AY538675	*α-CT*	alpha-carboxyltransferase	F:5'CTTGTCCACCCTATTCTGATTG3'	106	0.958
				R:5'ATGTCCAGCTTAGATTTGAGGC3'		
	Z50868	*β-CT*	Beta-carboxyltransferase	F:5'CAGCAAGTTTGGGTATGTTGGG 3'	116	1.030
				R:5'GTGAACCTTCAGGCACGGCTTT3'		
	AY034410	*BC*	Biotin carboxylase	F:5'AGGACCCATTCAAAGGATTCAG3'	118	1.000
				R:5'GCTTGGAGGAACAACATAGTCG3'		
Desaturase	AY642537	*SAD*	Stearoyl-ACP desaturase	F:5'GTTTACACTGCCAAAGACTATGCG3'	135	0.937
				R:5'CCTGATTCTCGGAGTCAACCCAC3'		
	AY592975	*FAD2*	Oleate desaturase	F:5'AGGCGATAAAGCCGATACTTGG3'	107	1.095
				R:5'CCTATCCGGTTCAACATAGATACACT3'		
	AY599884	*FAD3*	Linoleate desaturase	F:5'TTCCCACAAATCCCTCACTATCA3'	132	0.936
				R:5'ACTTGCCACCAAACTTTCCACC3'		
	AY642535	*FAD6*	Oleate desaturase	F:5'ATCACATAAGCCCAAGGATACCG3'	116	0.953
				R:5'TCGTCTTCATCAACCGCCAATT3'		
Elongase	AJ007046	*MCAT*	Malonyl CoA-acyl carrier protein transacylase	F:5'ATCATAGGGTTGGACTCAGAAA 3'	116	0.955
				R:5'ACTGCGTAGTTACCCGGACATA 3'		
	AF244519	*KAS1*	Beta-ketoacyl-ACP synthase 1	F:5'ACACGGTCGCAAACGAGAAGAA3'	204	0.976
				R:5'GAAGATAATGGTGATGGAGCAG3'		
	AF244520	*KAS2*	Beta-ketoacyl-ACP synthase 2	F:5'GGAGTACCAAGCCCTTGCTCAC3'	133	0.812
				R:5'TCCTTATGGCCTGCACAGTTGC3'		
	AF179854	*KAS3*	Beta-ketoacyl-ACP synthase 3	F:5'GGATGATGGGTTATTTAGTTTC3'	108	0.918
				R:5'CCAAAGGGTAAAGCAGGAGAAG3'		
	AF009563	*FAE1*	Fatty acid elongase 1	F:5'GTCAGGCTTTAAGTGTAACAGTGCA3'	159	0.957
				R:5'TTATTAGGACCGACCGTTTTGG3'		
	AF382146	*KACD*	3-keto-acyl-acp dehydratase	F:5'GATAGCGAAAATGGAAGGGAAAG3'	115	0.958
				R:5'AAAGCAAAAGGCACGAGAACATA3'		
	AY196197	*KCR2*	3-ketoacyl-CoA reductase	F:5'TGAGTACAAGAAAAGTGGGATTG3'	101	0.983
				R:5'GAGATGCCACTAAGAAAGATGCT3'		
Thioesterase	BRU17098	*FatA*	Acyl-ACP thioesterase	F:5'GGGACCAATGGCTCTGCATCAT3'	121	0.965
				R:5'GGCTTCTTTCTCCACAGGGTTG3'		
	DQ847275	*FatB*	Palmitoyl-ACP thioesterase	F:5'AGTTTGTGGGTGATGATGAATA3'	107	0.944
				R:5'GCAAGGATAGGGTCAGAGTTCA3'		
TAG synthesis	AF155224	*DGAT2*	Acyl-CoA: diacylglycerol acyltransferase	F:5'CATGACCTGATGAACCGCAAAG3'	111	0.985
				R:5'ACGGCTACCAAAAGGATACAAAA3'		
	AF111161	*LPAAT*	Plastidial lysophosphatidic acid acyltransferase	F:5' CGAAGAGGCGAGAAACAAGATAG3'	100	0.970
				R:5'TGGTTTAGCCTTCTCATTGTTCA3'		
	AY179560	*AAPT1*	Aminoalcoholphosphotransferase	F:5'TGGTGCTTCTTGGTTATTGTAT3'	156	0.821
				F:5'GACCCTCGATGGTGAGTTTGA3'		
Oil body protein	AY570250	*Napin*	1.7S oil body protein	R:5'CTTTGGATGCTCCTTTCAAGGT3'	148	0.958
				F:5'GTAATCAATTTGGCCCTTAGCT3'		
	AY966447	*Caleosin*	Ca2+-binding oil body surface protein	R:5'CTCAAGATTCACAGGCATAAAC3'	116	0.952
				F:5'CTGGGAGGCAAAGTTCAGGATA3'		
	X58000	*Oleosin*	oil body associated protein	R:5'CATGGCGTAATTTAGGTAGTGT3'	122	0.969
				F:5'GAGGAGTCAGAGACCGCAGGA3'		
	M16860	*Cruciferin*	12S neutral oil bodyprotein	R:5'AAGGAAGCGAAGGATGGGGAGA3'	165	0.969
				R:5'GGATTTGCATTATCCTCCCTTG3'		
Housekeep genes	AF111812	*β-actin*	Housekeeping gene	F:5'CTGGAATTGCTGACCGTATGAG 3'	145	1.001
				R:5'ATCTGTTGGAAAGTGCTGAGGG 3'		
	DQ097338	*GAPDH*	Glyceraldehyde-3-phosphate dehydrogenase	F:5'GCTATCAAGGAGGAATCTGAGGAC3'	146	0.936
				R:5'CTTCACGAAATTGTCACTCAACG3'		
Others	DQ167182	*P*450	Cytochrome P450	F:5'ATGGATCTCGGGATCGGACAGT3'	156	0.954
				R:5'GTCAAGCGATGACGGAGCAAAA3'		
	X93015	*GKTP*	Glyoxysomal beta-ketoacyl-thiolase precursor	F:5'GTTGGTCCAGCAGTTGCCATTC3'	159	0.934
				R:5'CGCCTCCGTTGACATTGATTTT3'		
	AJ223497	*PEPC*	Phosphoenolpyruvate carboxylase	F:5'GGTTGGGTTTATTGGTTTGTTTATG3'	134	0.934
				R:5'ATTCCCTTGCTCGGTTTTGTTA3'		
	AJ271162	*AGPase*	ADP-glucose pyrophosphorylase small subunit	F:5'AGACACCACCACCCCGTTTGAC3'	129	0.974
				R:5' TTTAGGGATAAGGCAGGAGGAT3'		
	AB041622	*BcRK6*	Receptor kinase 6	F:5'AGGTTAAGTGACGGGCAAGAAA3'	143	1.019
				R:5'TTGAACGCAACAGCCAAGAAGT3'		
	AY065839	*SUC1*	Sucrose transporter	F:5'GCCAAGGACTGTCGTTAGGAGTTT3'	133	0.970
				R:5'TGCGATTGCTCCGACTATAAATG3'		

### Determination of Gene Transcript Levels

The transcript levels of genes were analyzed by quantitative reverse transcription (qRT-PCR). Total RNA was extracted according to the protocol described for *Arabidopsis *seed [59]. RNA pellets were dissolved in DEPC-treated water, quantified by absorbance at 260 nm and checked for quality by agarose gel electrophoresis. Total RNA samples were reverse transcripted to first-strand cDNA using an Oligo(dT)20 primer and ReverTra Ace-α-TM (TOYOBO Inc., Tokyo, Japan). The cDNA products were stored at -70°C.

Primers were designed with the Primer Premier 5.0 software (Premier Biosoft International Palo Alto, CA) in order to produce PCR products that were 100–160 bps in size and located near the 3' UTR (Table 4). Optimized primer sets that produced no dimers and showed nearly 100% amplification efficiency were chosen. Information on optimized primer pairs is given in Table 4. The optimized conditions allowed for simultaneous analysis of multiple genes in a 96-well plate.

Real-time PCR reactions were performed in triplicate using the SYBR Green PCR Master Mix (TOYOBO Inc., Tokyo, Japan). A standard reaction mixture (20 μL) contained 1 μL cDNA template, 2× SYBR Green I Master Mix (TOYOBO Inc., Tokyo, Japan) and 400 nM forward and reverse primers.

The Opticon Monitor 2.0 (MJ Research) was used for all amplifications. The PCR protocol consisted of an initial denaturalization step at 94°C for 2 min, followed by 50 repeats of 94°C for 10 sec, 57°C for 20 sec and 72°C for 30 sec. PCR product specificity was confirmed by melting-curve analysis and by electrophoresis on 2% ethidium bromide-containing agarose gels to ensure that PCR reactions were free from primer dimers and non-specific amplicons.

For each gene, triplicate sets of PCR reaction samples were prepared and run in a 96-well plate. The PCR experiments were repeated for each plate to ensure that similar results were obtained. SYBR Green fluorescence was analyzed by Opticon Monitor 2.0 (MJ Research) software and the CT value for each sample was recorded for further analysis.

### Determination of PCR Efficiency for the 32 Genes

PCR efficiency was assayed for each gene. Plasmid DNA containing the gene of study was diluted in 5 serial ten-fold dilutions. An XY (scatter) plot was drawn and the linear regression equation was developed with the log input for the X values and the CT (Cycle of threshold) for the Y values. The results are listed in Table 4. The regression slope was used to calculate the PCR efficiency according to the following equation.



### Quantitative RT-PCR Data Analysis

The relative expression ratio of each gene compared with the control gene *β-actin *was determined using the Pfaffl method [60] and defined as Transcript Level (TL) in this paper. The relative expression ratio is calculated only from the real-time PCR efficiencies and the crossing point deviation of an unknown sample versus a control [60]. This model needs no calibration curve. Control levels were included to standardize each reaction run with respect to RNA integrity, sample loading and inter-PCR variations [61,62].

The CT values recorded in the Opticon Monitor 2.0 (MJ Research) were downloaded and processed in EXCEL. The mean CT value for each gene was calculated from three replicates and used for further calculations of the Transcript Level (TL) using the following formula [60]:



in which E_ref _is the PCR efficiency of the control gene *β-actin*, E_target _is the PCR efficiency of the gene under study, CT_ref _is the CT value of the control gene *β-actin*, and CT_target _is the CT value of the gene under study. The TL value determined by this formula represents a relative abundance of the transcripted copy number of a gene and allows us to make comparisons between the transcript abundances for different genes and between two cultivars for a specific gene.

### GC Analysis

The dissected seeds were dried by refrigeration until their weight remained constant and then the dried seeds were weighed and finely ground in tubes. After dissolution in 1 mL petroleum ether/aether (v/v = 1:1), each sample was saponified in 1 mL 0.4 N methanolic-KOH (10% (w/v) KOH, 5% (v/v) H_2_O in methanol) at 60°C for 1 h. After saponification, samples were cooled on ice and added to 500 μg methy-nonadecanoate (C19:0) and 2 mL ddH_2_O, and vortexed. After centrifugation, the supernatant was analyzed by GC (Gas Chromatograph).

GC analysis was performed with a gas chromatogram (Agilent 5890N) fitted with a 30 m FFAP capillary column (ID 0.25 mm narrow bore and film thickness 0.5 μm). The GC conditions were set as follows: injection volume, 1 μL; injector temperature and flame ionization detector temperature, 260°C; carrier gas, nitrogen at a split vent ratio of 50:1; column head pressure, 172.7 kPa (25 psi) to make up a total flow of 78.2 mL/min and a column flow of 1.5 mL/min (average velocity, 42 cm/s); hydrogen flow, 40 mL/min; air flow, 400 mL/min; running temperature program, 160°C for 1 min, and then increased 4°C per min to 240°C and held at this temperature for 10 min.

The result of GC analysis is displayed by its absolute value and relative value. The absolute value and relative value were calculated by



where A_i _is the area of the fatty acid peak, A_s _is the area of the control peak, m_s _is the mass quantity of the control, and m is the dry weight of the sample. Every sample was analyzed three times. The average value and standard deviation were calculated for each sample.

### Statistical Analysis

As shown in Table 2, the mean (Mean TL), coefficient of variation (C.V. of TL) and relative value of the transcript levels (RTL) were determined in order to characterize the transcript abundance of the 32 genes in study over time.

The mean transcript level (Mean TL) for each gene was calculated as an average of 8 stages from 10 to 45 DAP, and the relative transcript level (RTL) was calculated as the mean transcript level of the gene divided by the sum of the mean transcript levels of 32 genes and expressed as a percentage. The coefficient of variation (C.V. of TL) was expressed as the standard deviation as a percentage of the mean to measure the variation in transcript abundance of a gene during the time course.

A correlation analysis was made between the fatty acid accumulation pattern and the transcript level time course for each gene using the software embedded in EXCEL (Table 3).

## Competing interests

The authors declare that they have no competing interests.

## Authors' contributions

CML conceived the study and edited the manuscript. YPH performed experiments and analyses and wrote the manuscript. GW and XDL performed validating experiments and analyses. YLC, YHW and LX contributed to the writing of the manuscript and to the experimental design. All authors read and approved the final manuscript.
